# Investigating the efficacy of osimertinib and crizotinib in phase 3 clinical trials on anti-cancer treatment-induced cardiotoxicity: are real-world studies the way forward?

**DOI:** 10.1177/10781552221077417

**Published:** 2022-02-15

**Authors:** Hasan Kobat, Islam Elkonaissi, Emma Foreman, Mary O’Brien, Mehmet Tevfik Dorak, Shereen Nabhani-Gebara

**Affiliations:** 1 Department of Pharmacy, School of Life Sciences, Pharmacy and Chemistry, 4264 Kingston University, London, Kingston Upon Thames, KT1 2EE, UK; 2 Pharmacy Department, 2153 Cambridge University Hospitals NHS Foundation Trus, Cambridge, Cambridgeshire, United Kingdom of Great Britain and Northern Ireland; 3 Pharmacy Department, 4970 Royal Marsden NHS Foundation Trust, London, UK; 4 Imperial College London, 4970 Royal Marsden NHS Foundation Trust, UK; 5 Head of School of Life Sciences, Pharmacy and Chemistry, 4264 Kingston University, London, Kingston Upon Thames, UK

**Keywords:** Cardiotoxicity, real-world evidence, real-world experience, anti-cancer treatment, cardio-oncology

## Abstract

**Background.:**

Oncology clinical trials demonstrate the risk of cardiotoxicity but are not sufficient to reveal the true risk. In this article, we compared the incidence of cardiotoxicity of crizotinib and osimertinib from a real-world study to data reported by phase 3 clinical trials.

**Methods.:**

Data from an ongoing real-world lung cancer study was used as a comparator. Patients were recruited retrospectively with the criteria of being diagnosed with non-small cell lung cancer and having received at least a course of treatment of tyrosine-kinase inhibitor and/or immune check-point inhibitor. Characteristics of the patients who developed cardiotoxicity associated with osimertinib and crizotinib in the real-world lung cancer study were analysed against the inclusion criteria of the corresponding phase 3 clinical trials. Variations of cardiotoxicity incidence among the real-world lung cancer study and clinical trials were investigated.

**Results.:**

18%, n = 37/206, of the patients developed cardiotoxicity. QTc prolongation was the most frequently observed cardiotoxicity (n = 12/37). Osimertinib and crizotinib were the most cardiotoxic agents, each responsible for seven cases of cardiotoxicity. FLAURA, AURA3, PROFILE 1007 and PROFILE 1014 were the included clinical trials for analysis. None of the patients who developed cardiotoxicity in the real-world study would have been eligible to participate in FLAURA and PROFILE 1014 study whereas n = 4/7 and n = 5/7 patients were eligible to participate in AURA3 and PROFILE 1007 trials, respectively.

**Conclusion.:**

Although phase 3 clinical trials play an important role in understanding the effectiveness and give insights on side-effect profiles, real-world studies can show the real risk of cardiotoxicity more accurately and realistically.

## Introduction

Lung cancer is the second most frequently diagnosed type of cancer, accounting for 11.4% of all cancer cases.^1^ The introduction of novel therapies, such as tyrosine-kinase inhibitors (TKIs) and immune check-point inhibitors (ICIs) has resulted in improved efficacy over traditional chemotherapy in terms of progression-free survival and improved adverse events profile.^2^ Although the use of targeted therapies and immunotherapy are well tolerated by patients, side effects restrict the benefits. These include skin toxicities, haematological problems, vomiting, nausea, diarrhoea, oedema, thyroid gland related side-effects and cardiotoxicity.^3–5^ Cardiac toxicity-induced by anti-cancer agents is a serious adverse event and was extensively studied in breast cancer patients whose therapeutic regimen includes anthracycline group of agents and trastuzumab. As the number of TKIs and ICIs is growing, cardiotoxicity has become a concern with these agents. QTc prolongation, thromboembolic events, heart failure, left ventricular systolic dysfunction, life-threatening arrhythmias, hypertension, and bradycardia are some of the cardiovascular side effects related to TKIs and ICIs.^6^

Randomised controlled trials (RCTs) are a significant part of drug approvals. Survival, efficacy, tolerability, and side effects can be investigated via clinical trials. Several limitations; however, such as the duration of a trial and strict eligibility criteria restrict findings on the true risk of cardiotoxicity-induced by anti-cancer treatments. Real-world studies are a valuable part of clinical research, filling the gaps that cannot be provided by clinical trials, through describing the disease burden, incidence rates, mortality, and side effects to a greater degree.

In this article, we aim to compare the development of cardiotoxicity reported in our real-world lung cancer study to those in published RCTs. We also investigated whether the patients who developed cardiotoxicity associated with osimertinib and crizotinib in our ongoing real-world lung cancer study would have met the inclusion criteria in phase 3 clinical trials.

## Methods

### Real-world lung cancer study: design, eligibility, and data collection procedures

This retrospective real-world lung cancer study was approved by The Royal Marsden NHS Foundation Trust, a specialist cancer hospital in London, United Kingdom. A multi-disciplinary team including an oncologist, pharmacists, and academics designed the study protocol.

Inclusion criteria include a diagnosis of non-small cell lung cancer (NSCLC) having been treated with at least one dose of an anti-cancer agent including TKI and/or ICIs. Patients treated with ‘traditional chemotherapy only’ were excluded. Administration of chemotherapy following TKIs, and ICIs was acceptable. The data was retrospectively collected via hospital electronic patient records using a pre-specified data collection sheet.

Cardiotoxicity was defined as per the Common Terminology Criteria for Adverse Events (CTCAE) v5.^7^ Severe forms of several electrocardiography (ECG) and echocardiography changes such as atrial ectopic beats, left ventricular hypertrophy and diastolic function impairment were also considered as cardiotoxicity. Suspected patients from a cardiotoxicity point of view were detected by pharmacists but the final decision of drug-induced cardiotoxicity development was made upon review of suspected patients by the multi-disciplinary team.

The data collection sheet was made up of three different sections. The first section contains personal information and patient background: date of birth; gender; family history of cardiovascular disease; smoking status; alcohol use; profession; baseline comorbidities; baseline height and weight; ethnicity; allergies; and drug history. The second section includes information about the tumour, diagnosis, and anti-cancer treatment. This section includes: the date of diagnosis; histopathological diagnosis; stage; tumour wildtype; anti-cancer treatment; radiotherapy; prior exposure to anti-cancer therapy; and radiation. The third section is patient monitoring. Any cardiovascular checks that have been taken place such as ECG; echocardiography; physical cardiology checks; blood pressure; and heart rate measurements was recorder in this section.

### Search strategy, detecting clinical trials and comparison

Osimertinib and crizotinib phase 3 NSCLC clinical trials that were published in academic journals were the articles of interest ‘Osimertinib’, ‘crizotinib’, ‘clinical trial’, ‘phase 3’, ‘non-small cell lung cancer’ and their variations were the keywords used for the advanced literature search via PubMed and ScienceDirect to discover studies. Eligible studies were double checked through ClinicalTrials.gov by using their registry number provided within the published version. Phase 3 clinical trials and the real-world lung cancer study were then compared in terms of study protocols, incidence and types of cardiotoxicity.

## Results

### Baseline characteristics of the patients in the real-world lung cancer study

To date, 517 patients' case notes have been reviewed for eligibility. N = 206/517 met the inclusion criteria. 18%, n = 37/206, of the patients developed cardiotoxicity associated with anti-cancer treatment use. The mean age of patients with and without cardiotoxicity was 60 years (range: 34–81 years) and 65 years (range: 30–92). 46%, n = 17/37, of the patients with cardiotoxicity and 63.3%, n = 107/169, without cardiotoxicity had a history of smoking. 32.4%, n = 12/37 of the patients in cardiotoxicity group had at least one cardiovascular comorbidity whereas n = 7/37, 18.9%, of them had cardiac disease prior to treatment. Presence of cardiovascular comorbidities were more prevalent in patients without cardiotoxicity (47.9%, n = 81/169) whereas pre-existing cardiac disease was less prevalent (11.8%, n = 20/169). Hypertension was the predominant cardiovascular comorbidity for both groups (with cardiotoxicity: 27%, n = 10/37 vs. without cardiotoxicity: 33.1%, n = 56/169). Ischaemic heart disease was the most common type of cardiovascular disease prior to anti-cancer therapy in patients with and without cardiotoxicity (cardiotoxicity: 10.8%, n = 4/37 vs. no cardiotoxicity: 8.9%, n = 15/169). Detailed baseline characteristics of the patients with and without cardiotoxicity are listed in Table 1.

**Table 1.** Demonstration of baseline demographics of patients with or without cardiotoxicity (n = 206).

**Baseline characteristics**	**Cardiotoxicity (n = 37)** **Number of patients (%)**	**No cardiotoxicity (n = 169)** **Number of patients (%)**	*P-value
**Mean age, years (range)**	60 (34–81)	65 (30–92)	.03
**Female sex**	18 (48.7)	98 (58)	.36
**History of smoking**	17 (46)	107 (63.3)	.06
**Cardiovascular comorbidities**	12 (32.4)	81 (47.9)	.10
** Hypertension**	10 (27)	56 (33.1)	.56
** Type 2 diabetes**	5 (13.5)	11 (6.5)	.17
** Hypercholesterolemia**	5 (13.5)	19 (11.2)	.77
** Hypothyroidism**	2 (5.4)	13 (7.7)	1.00
** Acute kidney injury**	1 (2.7)	0 (0)	.18
** Type 1 diabetes**	0 (0)	6 (3.6)	.59
** Hyperthyroidism**	0 (0)	7 (4.1)	.36
** Chronic kidney disease**	0 (0)	3 (1.8)	1.00
**Previous cardiac disease**	5 (13.5)	20 (11.8)	.78
** Ischaemic heart disease**	4 (10.8)	15 (8.9)	.75
** Congestive heart failure**	0 (0)	1 (0.6)	1.00
** Atrial fibrillation**	1 (2.7)	9 (5.3)	.69

*All *P*-values are two-sided. t-test was used for the age and Fisher's exact test was used for categorical variables. All values are numbers and percentage in bracket unless otherwise specified.

### The types and frequencies of cardiovascular events (n = 37)

**Figure 1.**
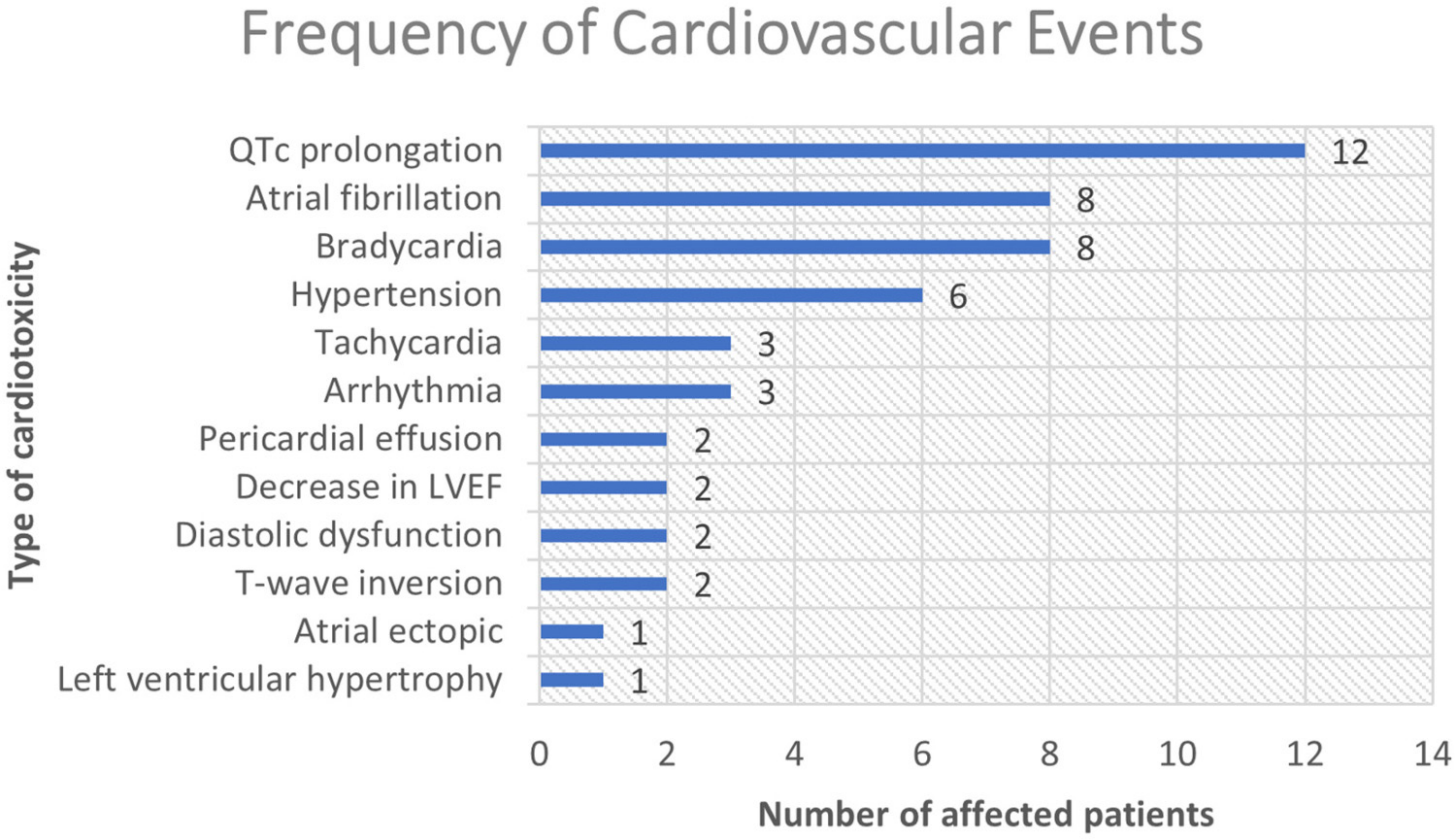
Demonstration of different cardiovascular events and the number patients who were affected. LVEF: Left ventricular ejection fraction.

**Figure 2.**
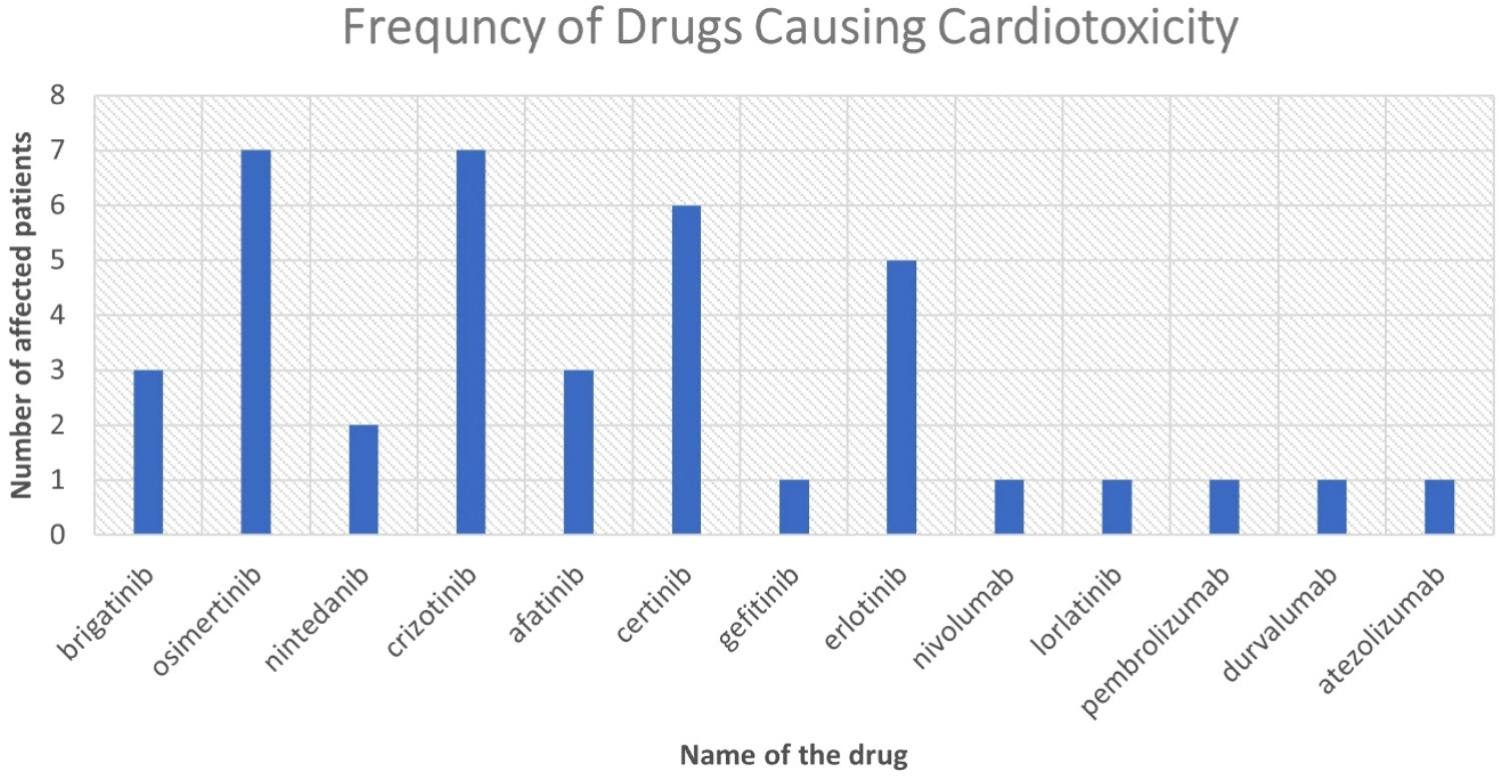
Demonstration of anti-cancer drugs which were responsible for the cardiac events. Two patients developed cardiotoxicity due ceritinib after they exhibited different type of cardiotoxicity with crizotinib.

Patients were followed-up from the date of NSCLC diagnosis until the availability of the last case note within the hospital electronic record. Majority of the patients received more than one TKI and/or ICI throughout the therapy. Regimen changes were due to a low or no prognostic effect of the anti-cancer drug, disease progression, and/or side effects which restrict the use of medication. There were various forms of cardiotoxicity that affected 37 patients, with 14 different types of cardiovascular events being observed. The most predominant type of cardiotoxicity was QTc prolongation, which affected 12 out of 37 patients. This accounts for 32.4% of the total cardiovascular events (Figure 1). The second most common type of cardiac event was atrial fibrillation and bradycardia, each affecting eight patients. Drug-induced hypertension was observed in six patients. Tachycardia, arrhythmia, pericardial effusion, drop in left ventricular ejection fraction, diastolic dysfunction, T-wave inversion, atrial ectopic and left ventricular hypertrophy were other types of cardiotoxicities which were detected in ≤ 3 patients. Most of the patients developed only one cardiovascular event during and/or after the course treatment (n = 25/37). The rest developed two to four different cardiotoxicities. Figure 2 demonstrate the full list of drugs and the number of patients affected by cardiotoxicity incidence.

### Osimertinib-related cardiotoxicity: The types and frequencies

The highest number of cardiotoxicity cases were associated with osimertinib and crizotinib use, each associated with seven patients. N = 33/206, 7.3%, patients received osimertinib of which 21.2%, (n = 7/33), developed cardiotoxicity. While the mean age was 67.6 years (range:56–81 years) for the patients with cardiotoxicity, this was 64.7 years (range: 44–86 years) for the patients without cardiotoxicity (*P* = .58). Female sex was the highest for both groups. (cardiotoxicity: n = 4/7, 57.1% vs. no cardiotoxicity: n = 21/263, 80.8%, *P* = .32). Mean duration of exposure to osimertinib was 8.7 months (range: 1–20 months) in patients with cardiotoxicity while this was 14.6 months (range: 1–39 months) for the patients who did not exhibit any cardiotoxicity. Baseline ECG was carried out for all the patients as part of the local regimen protocol. The mean number of total ECGs evaluation at baseline (prior to starting osimertinib), during and post-osimertinib was 5.7 (range: 2–13) for the patients who developed cardiotoxicity whereas this was 4.5 (1–14) for those without cardiotoxicity. Baseline QTc interval was nearly the same for both groups (cardiotoxicity: 425 ms vs. no cardiotoxicity 430 ms, *P* = .51). N = 5/7, 71.4, patients were deceased at the end of the follow up in the cardiotoxicity group, whereas this was 15 out of 26, 57.7%, in patients who received at least a course of osimertinib and did not develop cardiotoxicity (*P* = .68). The duration of follow up starting from the date when osimertinib initiated was 16.3 months (range: 3–32 months) for the patients who developed cardiotoxicity, whereas this was 19.4 months (range: 1–44 months) for the patients without cardiotoxicity (*P* = .51) (Table 2).

**Table 2.** Clinical characteristics and follow-up data of the patients with and without osimertinib-induced cardiotoxicity.

**Patients received osimertinib (n = 33)**			
	**Cardiotoxicity** **Number of patients (%) (n = 7)**	**No cardiotoxicity** **Number of patients (%) (n = 26)**	*P-value
**Mean age, years (range)**	67.6 (56–81)	64.7 (44–86)	.58
**Female sex**	4 (57.1)	21 (80.8)	.32
**History of smoking**	1 (14.3)	11 (42.3)	.22
**Cardiovascular comorbidities**	3 (42.9)	10 (38.5)	1.00
**Previous cardiac disease**	0 (0)	1 (3.8)	1.00
**Exposure to osimertinib, mean months (range)**	8.7 (1–20)	14.6 (1–39)	.18
**Follow-up time after onset to osimertinib, mean months (range)**	16.3 (3–32)	19.4 (1–44)	.51
**Incidence of cardiotoxicity after onset to osimertinib, mean months (range)**	5.4 (1–14)	N/A	N/A
**Number of ECG assessments, mean (range)**	5.7 (2–13)	4.5 (1–14)	.46
**Baseline ECG**	7 (100)	26 (100)	1.00
**Baseline QTc interval, mean ms**	425 (401–446)	430 (397–470)	.51

*All *P*-values are two-sided. t test was used for the continuous variables and Fisher's exact test was used for categorical variables. ECG: electrocardiography. All values are numbers and percentage in bracket unless otherwise specified.

Incidence of cardiotoxicity-associated with osimertinib was developed at 5.4 months which ranged from 1 to 14 months from starting the osimertinib treatment. Three out of seven patients developed only QTc prolongation and one patient developed atrial ectopic. The other three patients experienced multiple cardiac events: one developed arrhythmia (bradycardia), QTc prolongation, atrial fibrillation and cardiac failure (left ventricular ejection fraction: 30%); one experienced, tachycardia, and atrial fibrillation and one experienced QTc prolongation and cardiac failure (left ventricular ejection fraction: 30–35%). 3 out of 7 patients who developed cardiotoxicity upon osimertinib had cardiovascular comorbidities at the baseline. The ongoing drug history of the patients for the treatments of comorbidities revealed that there was no relationship with new onset cardiotoxicity development.

### Crizotinib-related cardiotoxicity: The types and frequencies

N = 22/206, 10.7%, patients received at least a cycle of crizotinib. Of the 22 patients, 7 (31.8%) of them developed cardiotoxicity which was believed to be induced by crizotinib. There was not statistically significant difference in the baseline age of the patients with and without cardiotoxicity (cardiotoxicity: 50.7 years (range: 34–70) versus no cardiotoxicity: 56.7 year (35–83), *P* = .35). 57.1%, n = 4/7, and 33.3%, n = 5/15, patients with and without cardiotoxicity were female, respectively. The number of smokers was also same. None of the patients in cardiotoxicity group had cardiovascular comorbidities and previous cardiac disease, whereas n = 6/22, 40%, and n = 2/22, 13.3%, in patients without cardiotoxicity had cardiovascular comorbidities and previous cardiac disease, respectively. Patients who developed cardiotoxicity had higher exposure to crizotinib with 22.9 months versus 14.3 months. Total follow-up time starting from the onset of crizotinib was 62.9 months with a minimum of 18 months in patients with cardiotoxicity. This was 25.9 months for the patients who did not develop cardiotoxicity (*P* < .001). All the patients in both groups had their baseline ECG. ECG assessments were more frequent in patients with cardiotoxicity than patients without cardiotoxicity (14 vs. 6.5, *P* = .01). Baseline QTc interval prior to start crizotinib was nearly the same (cardiotoxicity: 419.9 ms (range: 394–446 ms) versus no cardiotoxicity 418.3 ms (range: 377–440 ms), *P* = .88) (Table 3).

**Table 3.** Clinical characteristics and follow-up data of the patients with and without crizotinib-induced cardiotoxicity.

Patients received crizotinib (n = 22)			
	Cardiotoxicity	No cardiotoxicity	*P-value
	Number of patients (%) (n = 7)	Number of patients (%) (n = 15)	
Mean age, years (range)	50.7 (34–70)	56.7 (35–83)	.35
Female sex	4 (57.1)	5 (33.3)	.38
History of smoking	4 (57.1)	5 (33.3)	.38
Cardiovascular comorbidities	0 (0)	6 (40)	.12
Previous cardiac disease	0 (0)	2 (13.3)	1.00
Exposure to crizotinib, mean months (range)	22.9 (8–42)	14.3 (2–31)	.12
Follow-up time after onset to crizotinib, mean months (range)	62.9 (18–87)	25.9 (2–54)	.0006
Incidence of cardiotoxicity after onset to crizotinib, mean months (range)	12.7 (1–38)	N/A	N/A
Number of ECG assessments, mean (range)	14 (7–28)	6.5 (1–15)	.01
Baseline ECG	7 (100)	15 (100)	1.00
Baseline QTc interval, mean ms	419.9 (394–446)	418.3 (377–440)	.88

*All *P*-values are two-sided. t test was used for the continuous variables and Fisher's exact test was used for categorical variables. ECG: electrocardiography. All values are numbers and percentage in bracket unless otherwise specified.

Cardiotoxicity was observed at 12.7 months ranging from 1 to 87 months. Five of the seven patients who developed cardiotoxicity exhibited only one event which was hypertension (n = 1) and bradycardia (n = 4). The other two patients developed multiple events. One experienced QTc prolongation and hypertension, while the second patient developed bradycardia, atrial fibrillation, and T-wave inversion. All cardiac events were new and occurred during or post-crizotinib without being exposed to other drugs post-crizotinib.

### Clinical trials and comparison with the real-world study

According to the search strategy, FLAURA and AURA3 phase 3 trials were included into analysis for osimertinib and PROFILE 1007 and PROFILE 1014 phase 3 trials for crizotinib.^2,8–10^

#### FLAURA

##### Study design, eligibility, outcomes and side effects

This double-blind phase 3 trial aim to divide the patients to two different trial groups (FLAURA Funded by AstraZeneca; ClinicalTrials.gov number, NCT02296125).^2^ 279 patients were assigned to receive osimertinib while the rest standard *EGFR*-TKI (Table 4). The target population to test the efficacy and the safety of osimertinib were the NSCLC patients. Tumour evaluations were carried out prior to treatment, every six weeks for 18 months, then every 12 weeks until there is an evidence of disease progression. Unlike our real-world lung cancer study, the FLAURA trial had strict eligibility requirements. Only treatment-naïve patients were eligible to participate. Patients who currently suffer from various pre-existing conditions such as malignancies that need systemic therapy were excluded. Evidence of some cardiac abnormalities, *e.g.*, long QT syndrome, were also considered as exclusion criteria. Detailed exclusion standards were listed in Table 5. The median time for the treatment exposure was 16.2 months for osimertinib group and 11.5 months for standard *EGFR*-TKI group. Disease progression or death was less incident in the osimertinib group and the progression-free survival with osimertinib was significantly better. Rash or acne and diarrhoea were the most frequently observed adverse events for both groups that demonstrated for more than 50% of the participants of both groups. Patients who exhibited adverse event of ≥ 3 were less in osimertinib group (34%) than in standard *EGFR*-TKI group (45%). 10.4% of the patients developed prolongation in QT interval in the osimertinib group, whereas this was 4% for the standard *EGFR*-TKI group. Dose interruptions and reductions were observed in nearly 18% of the patients who developed QT prolongation in the osimertinib group. Fatal cases of QT prolongation and torsades de pointes were absent. Six patients in osimertinib group developed QT prolongation of grade ≥ 3 whereas only two patients in *EGFR*-TKI group had the same grade QT prolongation. The total number of patients who experienced a decrease in left ventricular ejection fraction was 18 out of 279, 6.5%, which represents >10% decrease together with a drop to a level <50%. 12 cardiac failure cases were also reported in osimertinib group mainly associated with left ventricular ejection fraction decrease (n = 10/12) (Table 6). Atrial fibrillation, myocardial infarction, angina pectoris, cardiac tamponade, tachyarrhythmia, hypertension and cardiac arrest were other cardiotoxicities observed less than other events. Recently, study sponsor, AstraZeneca has updated the side effects in ClinicalTrials.gov which were included in the Table 6.

**Table 4.** Full description of osimertinib and crizotinib phase III clinical trials (i.e., FLAURA, AURA3, PROFILE 1007 and PROFILE 1014.

**Osimertinib and crizotinib phase III clinical trials**				
	**FLAURA**	**AURA3**	**PROFILE 1007**	**PROFILE 1014**
**Target drug**	**Osimertinib**	**Osimertinib**	**Crizotinib**	**Crizotinib**
**Study design**	- Patients were assigned 1:1 ratio to receive osimertinib or standard *EGFR*-TKI (erlotinib or gefitinib). - Osimertinib dose: 80 mg daily vs 250 mg daily gefitinib or 150 mg daily erlotinib. - End-points: progression-free survival, overall survival, response rates and safety and patient-reported outcomes. - Patient recruitment was from December 2014 to March 2016 and the data cut-off date was June 2017. Max. follow up: 30 months.	- Patients were assigned 2:1 ratio to receive osimertinib or cisplatin/carboplatin + pemetrexed. - Osimertinib dose: 80 mg daily vs cisplatin dose: 75 mg/m^2^ or carboplatin dose: AUC5 + pemetrexed dose: 500 mg/m^2^ of BSA. - End-points: progression-free survival, overall survival, response rates and safety and patient-reported outcomes. - Patient recruitment was from August 2014 to September 2015 and the data cut-off date was April 2016. Max. follow up: 20 months.	- Patients were assigned 1:1 ratio to receive crizotinib or chemotherapy (*i.e.* pemetrexed or docetaxel) - Crizotinib dose: 250 mg twice daily vs pemetrexed dose: 500 mg/m^2^ of BSA or docetaxel dose. - End-points: progression-free survival, overall survival, response rates and safety and patient-reported outcomes. - Patient recruitment was from February 2010 to February 2012 and the data cut-off date was March 2012. Max. follow up: 25 months.	- Patients were assigned 1:1 ratio to receive crizotinib or cisplatin/carboplatin + pemetrexed. - Crizotinib doe: 250 mg twice daily vs cisplatin dose: 75 mg/m^2^ or carboplatin dose: AUC5/6 + pemetrexed dose: 500 mg/m^2^ of BSA. - End-points: progression-free survival, overall survival, response rates and safety and patient-reported outcomes. - Patient recruitment was from January 2011 to July 2013 and the data cut-off date was November 2013. Max. follow up: 29 months.
**Eligibility criteria**	- Treatment-naïve patients. - >18 years old. - Stage IIIB or IV NSCLC. - Patients with certain pre-existing conditions were excluded. Detailed exclusion criteria were listed in Table 5.	- Patients previously treated with other first-line *EGFR*-TKI. - >18 years old. - Stage IIIB or IV NSCLC. - Patients with certain pre-existing conditions were excluded. Detailed exclusion criteria were listed in Table 5.	- Patients previously treated with platinum-based therapy. - >18 years old. - Stage IIIB or IV NSCLC. - Patients with certain pre-existing conditions were excluded. Detailed exclusion criteria were listed in Table 5.	- Treatment-naïve patients. - >18 years old. - Stage IIIB or IV NSCLC. - Patients with certain pre-existing conditions were excluded. Detailed exclusion criteria were listed in Table 5.
**Outcomes**	- Total sample size: 556 (osimertinib arm: 279 vs. *EGFR*-TKI arm: 277). - Median treatment time = osimertinib: 16.2 months vs *EGFR*-TKI: 11.5 months. - Disease progression = osimertinib: 49%, n = 136/279 vs *EGFR*-TKI: 74%, n = 206/277. - Progression-free survival = osimertinib: 18.9 months, 95% CI, 15.2 to 21.4 vs *EGFR*-TKI: 10.2 months, 95% CI, 9.6 to 11.1. - Median duration of response = osimertinib: 17.2 months vs. *EGFR*-TKI: 8.5 months.	- Total sample size: 419 (osimertinib arm: 279 vs chemotherapy arm: 140). - Median treatment time = osimertinib: 8.1 months vs chemotherapy: 4.2 months - Disease progression = osimertinib: 45%, n = 88/197 vs chemotherapy: 82%, n = 36/44. - Median progression-free survival = osimertinib: 10.1 months vs chemotherapy: 4.4 months (HR: 0.30; 95% CI, 0.23 to 0.41). - Median duration of treatment = osimertinib: 8.1 months vs chemotherapy: 4.2 months.	- Total sample size: 347 (crizotinib arm: 173 vs chemotherapy arm: 174). - Median follow-up for overall survival = crizotinib: 12.2 months vs chemotherapy: 12.1 months - Disease progression was significantly lower with crizotinib (HR: 0.49; 95% CI, 0.37 to 0.64; P < 0.001). - Median progression-free survival = crizotinib: 7.7 (95% CI, 6.0 to 8.8) vs chemotherapy: 3 months (95% CI, 2.6 to 4.3). - Continuation of treatment at the data cut-off date = crizotinib: 49% vs chemotherapy: 16%.	- Total sample size: 343 (crizotinib arm: 172 vs chemotherapy arm: 171). - Median follow-up for overall survival = crizotinib: 17.4 months vs chemotherapy: 16.7 months. - Median progression-free survival = crizotinib: 10.9 months (95% CI, 8.3 to 13.9) vs chemotherapy: 7 months (95% CI, 6.8 to 8.2). - There was no significant difference in overall survival. - Objective response rate = crizotinib: 74% (95% CI, 67 to 81) vs chemotherapy: 45% (95% CI, 37 to 53) (*P* < .001)
**Side effects focusing on cardiotoxicity**	- QT prolongation = osimertinib: 10.4% vs *EGFR*-TKI: 4%. - Dose interruptions due to QTc prolongation in osimertinib arm: 18%. - QT prolongation of grade ≥ 3 = osimertinib: 6 patients vs *EGFR*-TKI group: 2 patients. - ≥ 10% drop in LVEF with osimertinib arm = 6.5%, n = 18/279. - Cardiac failure with osimertinib: 4.3%, n = 12/279. - Atrial fibrillation, myocardial infarction, angina pectoris, cardiac tamponade, tachyarrhythmia, hypertension and cardiac arrest were other cardiotoxicities observed less than other events.	- QT prolongation = osimertinib: 3.6%, n = 10/279 vs chemotherapy: 1 patient. - ≥ 10% drop in LVEF with osimertinib: 5%, n = 14/279. - Cardiac failure with osimertinib: 3.2%, n = 9/279.	- QT prolongation = crizotinib: 3.5%, n = 6/172. - 1 patient died of ventricular arrhythmia in crizotinib arm	- QT prolongation = crizotinib: 2.3%, n = 4/171. - Bradycardia, cardiac tamponade, atrial fibrillation, atrioventricular block were other cardiotoxicities observed.

AUC: area under the curve, BSA: body surface area, CI: confidence interval, EGFR: epidermal growth factor receptor, HR: hazard ratio, LVEF: Left ventricular ejection fraction, NSCLC: non-small cell lung cancer TKI: tyrosine-kinase inhibitor.

**Table 5.** General exclusion criteria of the phase III clinical trials (i.e. FLAURA, AURA3, PROFILE 1007 and PROFILE 1014).

**General exclusion standards of the phase III clinical trials**	
**Non-cardiovascular**	**Cardiovascular**
**Another ongoing therapeutic clinical trial**	Uncontrolled hypertension
**Spinal cord compression**	Prolonged mean resting QTc interval <470 ms or congenital long QT syndrome or family history of long QT syndrome or use of any medication that can prolong the QT interval
**Radiotherapy (<30%) to bone marrow**	Clinically significant deformities in conduction, rhythm, and morphology of ECG
**Patients receiving drugs that are potent inhibitor or inducer of CYP 3A4**	Complete left bundle branch block
**Any other concurrent malignancy that requires systemic treatment two to three years before the trial drug**	Second and third-degree heart block
**Unresolved toxicities associated with prior anti-cancer before the trial drug**	PR interval >250 ms in ECG
**Unstable and/or symptomatic brain metastasis**	Heart failure
**Any other diseases that are severe and/or uncontrollable**	Hypokalaemia
**Refractory nausea and vomiting**	Myocardial infarction
**Chronic gastrointestinal disease**	Unstable angina
**Cerebrovascular accident within three months prior to trial drug**	Coronary or peripheral artery bypass graft
**Pregnancy or breastfeeding.**	
**Any chronic psychiatric condition**	
**Chronic laboratory abnormalities and/or inadequate bone marrow reserves**	
**interstitial lung disease**	

CYP: cytochrome P450, ECG: electrocardiography.

**Table 6.** Types and incidence of cardiotoxicities-induced by osimertinib reported by FLAURA, AURA3 and the real-world study.

**Cardiotoxicity-associated with osimertinib**					
	**FLAURA study paper (n = 279)** **Number of patients (%)**	**ClinicalTrials.gov FLAURA (n = 279) last updated results on December 30, 2020** **Number of patients (%)** **^11^**	**AURA3 study paper (n = 279)** **Number of patients (%)**	**ClinicalTrials.gov AURA3 (n = 279) last updated results on February 4, 2021** **Number of patients (%)** **^12^**	**Real-world lung cancer study: osimertinib subgroup (n = 33)** **Number of patients (%)**
**QTc prolongation**	29 (10.4)	29 (10.4)	10 (3.6)	1 (0.36)	5 (15.2)
**Cardiac failure**	12 (4.3)	N/A	9 (3.2)	2 (0.72)	2 (6.1)
** Cardiac failure associated with LVEF decline**	10 (3.6)	N/A	6 (2.2)	N/A	2 (6.1)
**Decline in LVEF**	8 (2.9)	N/A	8 (2.9)	N/A	2 (6.1)
**Atrial ectopic**	N/A	N/A	N/A	N/A	1 (3)
**Bradycardia**	N/A	N/A	N/A	N/A	1 (3)
**Atrial fibrillation**	1 (0.36)	1 (0.36)	N/A	2 (0.72)	2 (6.1)
**Tachycardia**	N/A	N/A	N/A	N/A	1 (3)
**Acute myocardial infarction**	1 (0.36)	1 (0.36)	N/A	N/A	0 (0)
**Angina pectoris**	1 (0.36)	1 (0.36)	N/A	N/A	0 (0)
**Cardiac tamponade**	1 (0.36)	1 (0.36)	N/A	1 (0.36)	0 (0)
**Cardiac arrest**	1 (0.36)	1 (0.36)	N/A	N/A	0 (0)
**Myocardial infarction**	1 (0.36)	1 (0.36)	N/A	N/A	0 (0)
**Tachyarrhythmia**	1 (0.36)	1 (0.36)	N/A	N/A	0 (0)
**Hypertension**	1 (0.36)	15 (5.38)	N/A	9 (3.23)	0 (0)
**Pericardial effusion**	N/A	N/A	N/A	1 (0.36)	0 (0)
**Atrial thrombosis**	N/A	N/A	N/A	1 (0.36)	0 (0)

LVEF: Left ventricular ejection fraction. Decrease in LVEF defined as an absolute decrease of >10% associated with a decline <50%. All values are numbers and percentage in bracket unless otherwise specified.

##### Eligibility criteria comparison: real-world lung cancer study versus FLAURA trial

According to findings from the published trial papers, assuming patients developed only one type of cardiotoxicity, the incidence of cardiotoxicity in osimertinib arm for FLAURA was n = 57/279, 20.4% compared to 21.2% of the patients in the real-world study. This is approximately same ratio with the demonstrated events in FLAURA (OR: 1.04; 95% CI: 0.4 to 2.5, *P* = .09). None of the patients who developed osimertinib-induced cardiotoxicity (n = 7) in the real-world lung cancer study were eligible to participate in the FLAURA trial. All our seven patients received either traditional chemotherapy and/or *EGFR*-TKI, prior to osimertinib, specifically for locally advanced or metastatic NSCLC. Apart from previous anti-cancer treatment use as an exclusion factor, three patients had multiple reasons to be excluded from the study. These included radiotherapy >30% of the bone marrow prior to treatment, chronic gastrointestinal disease, use of drugs which prolong QT interval, and the existence of ischaemic heart disease (Table 7).

**Table 7.** Patient exclusion criteria in osimertinib phase 3 clinical trials.

Osimertinib phase 3 clinical trials							
	AURA3			FLAURA			
Patient number	Significant clinical cardiac abnormality and use of medication that prolong QT interval	Chronic gastrointestinal disease	Radiotherapy >30% of the bone marrow	Prior treatment with any SACT for locally advanced/metastatic NSCLC and/or *EGFR*-TKI	Chronic gastrointestinal disease	Significant clinical cardiac abnormality and use of medication that prolong QT interval	Radiotherapy >30% of the bone marrow
1								
2							
3							
4							
5							
6							
7							

N = 4/7 patients in real-world lung cancer study were eligible to participate in AURA3 trials whereas none of them fit to participate in FLAURA trial. Red boxes show the exclusion reason for AURA3 and green boxes for FLAURA. EGFR-TKI: Epidermal growth factor receptor-Tyrosine kinase inhibitor, SACT: Systemic anti-cancer treatment, NSCLC: Non-small cell lung cancer.

#### AURA3

##### Study design, eligibility, outcomes and side effects

AURA3 is another double-blind phase 3 clinical trial conducted to evaluate if osimertinib in previously treated NSCLC patients is more effective than platinum-based therapy (Funded by AstraZeneca; ClinicalTrials.gov number, NCT02151981) (Table 4).^8^ Patients with various pre-existing diseases, unresolved toxicities, several cardiac conditions, and use of a group of drugs for other medical conditions were excluded. Detailed exclusion criteria were listed in Table 5. 279 patients were assigned to receive osimertinib and 140 platinum-based treatment. Progression-free survival was longer in the osimertinib arm. The duration of progression-free survival was also higher with osimertinib group. 98%, n = 273/279, of the patients in the osimertinib group developed at least one adverse event. Diarrhoea (41%), rash (34%), dry skin (23%), and paronychia (22%) were the most frequently observed adverse events. There was also reported side-effects associated with the cardiovascular system. Left ventricular ejection fraction decrease more than 10% and to a level >50% (n = 14/279, 5%), cardiac failure (n = 9/279, 3.2%) and QT prolongation (n = 10/279, 3.6%) were observed in patients receiving osimertinib (Table 6). However, only one patient in platinum-pemetrexed group demonstrated QT prolongation. Overall, osimertinib-induced side effects which were led to discontinuation of the trial drug were at a lower rate than platinum-pemetrexed group (osimertinib group: n = 19/279, 7% vs. platinum-pemetrexed group: n = 14/140, 10%). Only four patients in osimertinib group and one in platinum-pemetrexed group had died caused by treatment-induced adverse event. Some of the events were not demonstrated in the published study paper but were listed in ClinicalTrials.gov or vice versa (Table 6).

##### Eligibility criteria comparison: real-world lung cancer study versus AURA3 trial

27 out of 279, 9.8%, patients in AURA3 trial developed cardiotoxicity in osimertinib subgroup. Cardiotoxicity development of the real-world lung cancer study was higher than the AURA3 trial (OR: 2.5; 95% CI: 1.0 to 6.3, *P* *=* .07). Only four out of seven patients who developed cardiotoxicity in the real-world lung cancer study were eligible to participate in the AURA3 phase 3 trial. All four patients did not have any serious medical condition to be excluded from the AURA3 trial. The rest of the patients (n = 3/7) were not eligible to participate due to several reasons. First patient had ischaemic heart disease and was using a drug which can cause QTc prolongation before the onset of osimertinib. Second patient demonstrated chronic gastrointestinal disease and third patient received radiation >30% of the bone marrow which were considered within exclusion criteria according to AURA3 trial (Table 7).

#### PROFILE 1007

##### Study design, eligibility, outcomes and side effects

PROFILE 1007 is a double-blind phase 3 clinical trial that assessed the efficacy and safety of crizotinib in NSCLC patients (Funded by Pfizer; ClinicalTrials.gov number, NCT00932893).^9^ Previously treated patients were scheduled to receive either crizotinib (n = 173) or chemotherapy (n = 174) (Table 4). Tumour assessments (brain and bone scanning) were carried out at the baseline and every six weeks until disease progression. Cardiac or non-cardiac pre-existing conditions such as spinal cord compression, unstable angina, myocardial infarction, congestive heart failure, etc. were considered under exclusion criteria. A detailed list of exclusion factors was listed in Table 5. Median follow-up time for overall survival was around 12 months in both groups at the time of data cut-off. Median progression-free survival was higher with crizotinib. Likewise, the response rate to the treatment was higher in the crizotinib group. Vision disorder, diarrhoea and nausea affected more than 50% of the patients who were treated with crizotinib. This is followed by vomiting (n = 80/172, 47%), constipation (n = 73/172, 42%) and elevated aminotransferases (n = 66/172, 38%). QT prolongation detected by ECG was the major cardiovascular side-effect which was observed only in the crizotinib group (crizotinib: n = 6/172, 3.5%) (Table 8). Three patients in crizotinib arm died because of treatment-associated side effect. Deaths were related to ventricular arrhythmia, interstitial lung disease and pneumonitis.^13,14^

**Table 8.** Types and incidence of cardiotoxicities-induced by crizotinib reported by PROFILE1007, PROFILE1014 and the real-world study.

**Cardiotoxicity-associated with crizotinib**					
	**PROFILE 1007 study paper (n = 172) Number of patients (%)**	**ClinicalTrials.gov PROFILE 1007 (n = 172) last updated results on January 2, 2017** **Number of patients (%)** **^13^**	**PROFILE 1014 (n = 171) study paper** **Number of patients (%)**	**ClinicalTrials.gov PROFILE 1014 (n = 171) last updated results on November 6, 2017** **Number of patients (%)** **^14^**	**Real-world lung cancer study: crizotinib subgroup (n = 22) Number of patients (%)**
**QTc prolongation**	6 (3.5)	10 (5.8)	4 (2.3)	11 (6.43)	1 (4.5)
**Ventricular arrhythmia**	1 (0.6)	N/A	N/A	N/A	0 (0)
**Bradycardia**	N/A	9 (5.23)	N/A	33 (46.5)	5 (22.7)
**Hypertension**	N/A	N/A	N/A	N/A	2 (9.1)
**Atrial fibrillation**	N/A	N/A	N/A	1 (0.58)	1 (4.5)
**Arrhythmia**	N/A	1 (0.58)	N/A	N/A	0 (0)
**T-wave inversion**	N/A	N/A	N/A	N/A	1 (4.5)
**Cardiac arrest**	1 (0.58)	1 (0.58)	0 (0)	0 (0)	0 (0)
**Pericardial effusion**	0 (0)	1 (0.58)	N/A	0 (0)	0 (0)
**Coronary artery disease**	N/A	1 (0.58)	N/A	N/A	0 (0)
**Myocardial ischaemia**	N/A	1 (0.58)	N/A	N/A	0 (0)
**Syncope**	N/A	1 (0.58)	N/A	0 (0)	0 (0)
**Atrioventricular block**	N/A	N/A	N/A	1 (0.58)	0 (0)
**Cardiac tamponade**	N/A	1 (0.58)	N/A	2 (1.17)	0 (0)

All values are numbers and percentage in bracket unless otherwise specified.

##### Eligibility criteria comparison: real-world lung cancer study versus PROFILE 1007 trial

According to study paper of PROFILE1007 trial, the number of patients who developed cardiotoxicity in crizotinib arm was n = 7/172, 4.1%. The total number patients who received crizotinib in the real-world lung cancer study was n = 22/206 in which 7 out of 22 (31.8%) of them developed cardiotoxicity. Cardiotoxicity development in our real-world lung cancer study was significantly higher when compared with PROFILE 1007 (OR: 11; 95% CI: 3.4 to 35.6, *P* < .001). Five out of seven patients who developed crizotinib-induced cardiotoxicity were eligible to participate in this study. All five patients received only one platinum-based chemotherapy for locally advanced or metastatic NSCLC prior to crizotinib initiation. They did not have any previous serious acute or chronic disease conditions. The other two patients (n = 2/7) were not eligible to participate in this clinical trial. One received multiple previous chemotherapy consisting of carboplatin, pemetrexed, docetaxel, and bevacizumab and had a history of interstitial pneumonia before crizotinib therapy as the secondary exclusion reason. Other patient who was excluded had Gilbert's syndrome and >2 times the upper limit of normal bilirubin level. >1.5 times of normal bilirubin levels were considered as an exclusion criterion in PROFILE 1007 trial (Table 9).

**Table 9.** Patient exclusion criteria in crizotinib phase 3 clinical trials.

Crizotinib phase 3 clinical trials						
	PROFILE 1007			PROFILE 1014		
Patient number	Interstitial lung disease	Gilbert's syndrome together with bilirubin level ≤1.5 × ULN	Multiple previous chemotherapy treatment	Previous treatment with standard chemotherapy	Interstitial lung disease	Gilbert's syndrome together with bilirubin level ≤1.5 × ULN
1						
2						
3						
4						
5						
6						
7						

Five patients in the real-world lung cancer study were eligible to participate in PROFILE 1007. However, none of the patients fit for the PROFILE 1014's eligibility criteria. ULN: upper limit of normal. Yellow boxes show the exclusion reason for PROFILE 1007 trial and blue boxes for PROFILE 1014.

#### PROFILE 1014

##### Study design, eligibility, outcomes and side effects

PROFILE 1014 is another randomised, open-label phase 3 clinical trial which was carried on locally advanced or metastatic NSCLC patients to assess the efficacy and safety of crizotinib (Funded by Pfizer; ClinicalTrials.gov number, NCT01154140).^10^ Patients were assigned to either receive crizotinib (n = 172) or traditional chemotherapy (n = 171) (Table 4). Tumour evaluations were carried out at the baseline, every six weeks during the treatment and every six weeks post-treatment until disease progression. Treatment-naïve patients were enrolled in the PROFILE 1014 trial. Exclusion standards were similar to PROFILE 1007. Patients with several conditions including, but not limited to, spinal cord compression, previous malignancies, and some other pre-existing cardiovascular condition such as uncontrolled atrial fibrillation, long QT syndrome, etc. were excluded. Detailed list of exclusion criteria was listed in Table 5. The median progression-free survival of the crizotinib group was superior to patients treated with standard chemotherapy. The objective response rate to treatment was higher in patients receiving crizotinib. The median duration of response was doubled with crizotinib than with chemotherapy (crizotinib: 11.3 months vs. chemotherapy: 5.3 months). Vision disorder was the most frequent adverse event observed in crizotinib group (n = 122/171, 71%). The most significant cardiac side effect was QT prolongation (n = 4/171, 2.3%) in patients treated with crizotinib (Table 8). Like in the PROFILE 1007 trial, there are variations in between ClinicalTrials.gov and published study paper (Table 8).

##### Eligibility criteria comparison: real-world lung cancer study versus PROFILE 1014 trial

According to PROFILE 1014, n = 4/171, 2.3%, patients developed cardiotoxicity. Incidence of cardiotoxicity in the real-world lung cancer study was significantly higher than PROFILE 1014 (OR: 19.5; 95% CI: 5.1 to 74.2, *P* *<* .001) trial. None of the patients who experienced crizotinib-induced cardiotoxicity were eligible to participate in the PROFILE 1014 trial. The primary reason for the exclusion is the availability of previous treatment with standard chemotherapy regimens. The patients who demonstrated Gilbert's syndrome and interstitial lung disease were not eligible to participate in the PROFILE 1014 trial as were in PROFILE 1007 (Table 9).

## Discussion

To our knowledge, this is the first study to compare the NSCLC clinical trials with real-world studies in terms of development of cardiotoxicity. Cardiotoxicity was more frequently observed when compared with some of the phase 3 clinical trials. The reason for the differences in cardiotoxicity incidence is believed to be affected by the significant variations between methodologies of the clinical trials and our real-world lung cancer study. Differences in study protocols, in terms of the ability to detect cardiotoxicity development, was mainly related to the entry requirements/eligibility criteria and follow-up times.

### Entry requirements/eligibility criteria

#### Effect of previous anti-cancer treatment in developing cardiotoxicity

Exposure to previous anti-cancer treatment, not long before the trial drugs, was one of the major exclusion criteria from the perspective of the patients in the real-world study. None of the patients who developed cardiotoxicity in the real-world lung cancer study were eligible to participated in FLAURA and PROFILE 1014 trials. Patients would have been excluded due to previous treatment with standard chemotherapy. The main similarity of the patient histories was being exposed to previous first-line treatment with platinum-based agents. There might be an increased incidence of cardiotoxicity in patients with a history of other anti-cancer therapies. According to the position paper of the European Society of Cardiology (ESC), previous anthracycline treatment substantially increases the chance of cardiotoxicity development of trastuzumab.^15^ Even though platinum-based agents are not as cardiotoxic as anthracyclines and trastuzumab, they are also associated with cardiotoxicity including, congestive heart failure, electrical activity changes, myocarditis, acute myocardial infarction, heart failure, etc..^11,16^ Sequential use of platinum-based agents with potentially cardiotoxic TKIs, such as osimertinib and crizotinib may be one of the causes behind the development of cardiotoxicity. The relationship between the standard chemotherapy regimens prior to TKIs must not be ignored, however it has not been revealed yet. It is not possible to ascertain the effect of previous chemotherapy in developing cardiotoxicity with phase 3 clinical trials that indicates the need for real-world studies.

#### Impact of medical history in cardiotoxicity development

Medical history or in other words presence of pre-existing diseases is another entry requirement issue which might have an effect on developing cardiotoxicity when patients receive potentially cardiotoxic TKIs. Significant cardiac abnormality, chronic gastrointestinal disease, interstitial lung disease, radiotherapy >30% of the bone marrow, and Gilbert's syndrome were the pre-existing diseases demonstrated by the patients who developed cardiotoxicity in the real-world lung cancer study. Presence of some of these medical histories can be considered as risk factors in developing cardiotoxicity. In a study by Clarson et al. interstitial lung disease was found to be independently associated with ischaemic heart disease.^12^ Due to respiratory failure, low oxygen levels in blood together with increased pressures in the right ventricle and pulmonary artery can cause heart failure. Not only anti-cancer drug alone but also pre-existing lung disease may also have an effect on cardiotoxicity development. The existence of cardiac disease is another exclusion factor for cancer clinical trials which were presented in the real-world lung cancer study. One patient in the AURA3 trial had ischaemic heart disease at the baseline and developed a different type of cardiovascular event associated with osimertinib use. Application of potentially cardiotoxic anti-cancer agents in patients having cardiac disease at the baseline may increase the chance of development of other cardiac events. Risk stratification at the baseline is a significant assessment strategy which helps to implement the cardiovascular evaluation during and after the treatment as well as in choosing the most appropriate anti-cancer drug to be used.

### Follow-up period

Cardiotoxicity of anthracycline group of agents are cumulative dose-dependent whereas trastuzumab cardiotoxicity manifests as dose independent and can develop at any time.^13,14^ However, onset time for cardiotoxicity with osimertinib and crizotinib has not been revealed yet. According to existing evidence available in the literature, cardiotoxicity-associated with osimertinib was presented by several case studies showing that the occurrence of the cardiac events at the third weeks, two, four and sixth months after starting a treatment with osimertinib.^17–20^ According to a study by Del Valle et al. 22 patients were followed for 18 months and four of them developed cardiotoxicity (*i.e.* bradycardia, QTc prolongation, complete heart block) however, the exact onset time for cardiotoxicity was not presented.^21^ The number of studies showing crizotinib and osimertinib-induced cardiotoxicity are limited. Onset time for cardiotoxicity with these agents is unknown and may occur weeks to years after the initiation of treatment. The mean follow-up time of the patients in the real-world lung cancer study who received osimertinib, regardless of cardiotoxicity development, was 18.8 months (range: 1–44 months) starting from the date of osimertinib onset. The maximum follow up of the osimertinib subgroup in the real-world study was 44 months which is quite higher than FLAURA and AURA3 trials. There is also a big difference in the follow-up data of the patients with or without cardiotoxicity in crizotinib subgroup. Mean follow-up of the patients with and without cardiotoxicity, starting with the initiation of crizotinib, were 62.9 months (range: 18–87 months) and 25.9 months (range: 2–54 months), respectively (Table 3). However, in PROFILE1007 and PROFILE1014 trials this was 25 and 29 months, respectively. Patients in the crizotinib and osimertinib phase 3 clinical trials were followed much less than our real-world study. The exact onset of cardiotoxicity with osimertinib and crizotinib is unknown as described with existing evidence. Our real-world study shows that the maximum exposure to osimertinib and crizotinib is 39 (Table 2) and 42 months (Table 3), respectively. Likewise, another real-world study also demonstrated long term responders to crizotinib with as much as 73 months.^21^ These evidence shows that the number of patients who respond well to osimertinib and crizotinib is considerable which causes longer exposure to these anti-cancer drugs. As the exposure time to these agents increases, the chance of cardiotoxicity development also increases. Although the onset of cardiotoxic events were not presented in phase 3 clinical trials, we believe that the one cause of difference between the incidence of cardiotoxicity in the real-world lung cancer study and phase 3 clinical trials was the variations in the follow-up times. To understand the real risk of cardiotoxicity, patients should be followed at the baseline, during and as well as years after the termination of anti-cancer drugs.

### Optimal management strategy and implications on practice: QTc prolongation

QTc prolongation was the most predominant cardiotoxicity observed in the real-world lung cancer study. Even more, QTc prolongation was the most frequently adverse event for the patients who received osimertinib. According to CTCAE v5., *i.e.,* the document demonstrated the adverse events, QTc prolongation categorised from grade 1 to 5. Grade 1 QTc prolongation means QTc interval being 450–480 ms. Grade 2 QTc prolongation is 481–500 ms, and ≥ 501 ms; >60 ms change from baseline defines grade 3. The difference in grade 4 QTc prolongation is its symptomatic feature which was defined as Torsade de pointes; polymorphic ventricular tachycardia; symptoms of serious arrhythmia together with prolonged QTc greater than 500 ms. QTc prolongation can lead to severe outcomes such as impaired ventricular repolarisations followed by Torsade de pointes and sudden cardiac death.^22^ As only grade 4 QTc prolongation is symptomatic, development of long QT syndrome is silent most of the time. The majority of the patients in our real-world study demonstrated QTc prolongation of grade 1 to 3 and only a few developed grade 4. This shows the clinical significance of ECG screening to save patients from the development of potential QTc prolongation before it is life-threatening. According to package insert of osimertinib, it is advised to monitor patients with ECG at the baseline and regularly during the treatment.^23^ Although ECG is advised to be in the regular assessment procedures, there is no available data in the frequency of ECG evaluation. Assessment criteria of some hospitals in the United Kingdom suggest carrying out ECG at the baseline and if clinically needed during the treatment.^24^ According to The Royal Marsden NHS Foundation Trust, where the real-world study took place, unpublished assessment criteria suggests carrying out ECG at the baseline and before each cycle. Overall, as QTc prolongation is most frequently subclinical and the major type of cardiotoxicity, baseline and serial assessment with ECG needs to be clarified to save lives. Although the majority of the patients in the study hospital were monitored at the baseline and before each cycle, ECG data is missing for some of them (Tables 2 and 3) which indicates there may be some undetected cases. Although it was expected to observe more serial ECG assessments in clinical trials, there was no clear information on the frequency of ECGs in phase 3 clinical trials and this restricted us to compare serial monitoring. We believe that the optimal management with ECG should be at the baseline and before each cycle unless QTc does not appear to exceed 480 ms. If QTc is greater than 480 ms and up to 500 ms, *i.e.,* grade 2 QTc prolongation, patients should be monitored more closely with more frequent ECG assessments. As advised in the package inserts, treatment needs to be withheld in case of QTc ≥ 500 ms until it recovers to a level less than 480 ms and permanently discontinue when a patient clinically exhibit grade 4 prolonged QTc.

### Importance of research in cardio-oncology

In the past, anti-cancer drugs causing cardiotoxicity were not abundant, and the inter-professional healthcare system was not developed to efficiently participate in the management of the patients. In the past, cardiotoxicity was limited to anthracyclines, trastuzumab, cyclophosphamide, etc. Not many years ago, the introduction of new agents, including targeted therapies (*e.g.* TKIs) and ICIs, were found to alter the cardiovascular system, sometimes with fatal outcomes. Increased incidence and types of cardiotoxicities were observed in clinical trials and clinical practice with the resultant emergence of cardio-oncology. This is a multi-disciplinary and inter-professional discipline created to manage cardiotoxicity in several ways. These include serial monitoring for early diagnosis, prevention, risk stratification, and early treatment.^10^ Management strategies can be carried out after a reliable insight obtained via research studies. Research is a significant part of cardio-oncology. Understanding underlying reasons (*i.e.* pre-existing conditions, risk-factors in developing cardiotoxicity), detecting incidence and types of cardiotoxicity with different agents can be elucidated with comprehensive research studies which can light the way to effectively apply management strategies.

### Clinical trials and real-world studies

Phase 3 clinical trials are considered part of these research activities. Their findings reflect the efficacy and safety of drugs. Especially for cardiotoxic anti-cancer agents, clinical trials play an important role in detecting the cardiac side-effects, as ECG and echocardiography evaluations are normally a part of their protocol. However, understanding the cardiovascular safety profile of anti-cancer agents is not possible with only clinical trials. Clinical trials target a specific group of people with strict eligibility criteria. Inclusion and exclusion standards were adjusted in a way to include patients who can continue the trial drug with less chance of dose disruptions and drug withdrawals. Age, gender, cardiovascular and non-cardiovascular comorbidities, chronic diseases, smoking, weight, etc. may have an effect of increased risk in developing cardiotoxicity when combined with potentially cardiotoxic anti-cancer agents. The eligibility criteria of clinical trials exclude several groups of patients. As described in the results, all clinical trials exclude patients with chronic diseases and cardiovascular abnormalities. For example, patients with uncontrolled hypertension may develop another type of cardiac dysfunction when combined with cardiotoxic anti-cancer therapeutics. Clinical trials are not sufficient on their own to reveal the cause behind the impact of patient medical history on cardiotoxicity development after exposed to anti-cancer drugs.

### Increasing demand for real-world studies

According to our PubMed advanced search with the keywords “real-world study” and “real-world experience”, the number of studies available in 2011 was 5427 which increased to 13,690 five years later in 2016. As of 2020, this is reached to 32,432 which indicates that the number of studies nearly doubles when compared to previous five years. Cardio-oncology studies show that only a group of people are affected by cardiotoxicity despite all receiving the same dose most of the time. The reason behind this mystery has not been solved yet. Scientists believe that there must be other underlying factors triggering cardiotoxicity development. This could be through genetics, risk factors, pre-existing diseases, or all. To tackle this challenge, genetic studies are ongoing to find novel polymorphisms that are responsible for cardiotoxicity incidence.^25^ Understanding the effects of risk factors and pre-existing diseases are significant that will reveal the cause behind the discrepancy between the several groups of patients who develop cardiotoxicity and others who survive many years without any cardiovascular symptoms. The gap in the literature can be elucidated with increased number of real-world studies. This is because real-world studies have less strict eligibility criteria which enables patient inclusion with wider diversity.

### Limitations

The major limitation of this study is the low sample size. In total, only 55 patients received osimertinib and crizotinib of whom n = 14/55 developed cardiotoxicity. The purpose of this study was to illuminate that patients with distinct medical histories may have been excluded in clinical trials yet developed cardiotoxicity in real-world practice. This study is one of its kind that compared the results of the real-world studies with clinical trials from a cardiotoxicity perspective at a single institution; however, multi-institutional and multi-national studies of a similar type are strongly required to provide more meaningful results with increased sample size. Another potential limitation of this study is the disparities in cardiovascular monitoring of the patients in the real-world lung cancer study. The most common type of cardiovascular assessment tool was with using ECG, but the frequency of ECG screenings was not standardised in the retrospective real-world lung cancer study. While some patients who received osimertinib and crizotinib were screened only one time with ECG during their treatment, the maximum number of ECG assessments have been observed to reach up to 28 times for some patients (Tables 2 and 3). Subclinical cardiotoxicity may have gone unnoticed in patients who had a low number of cardiovascular examinations. Differences in cardiovascular monitoring among the patients who participated in clinical trials and the real-world lung cancer study can also be considered as a limitation. Despite the fact that the frequency of cardiovascular examinations was reported in the real-world lung cancer study in detail, cardiovascular safety was not one of the primary objectives of phase III clinical trials. The detailed results of the cardiovascular evaluations were not presented in the published versions of the phase III clinical trials. Having said that, although some patients have more frequent cardiovascular examinations in the real-world lung cancer study, the real-world practice was not standardised like in the clinical trials. Another limitation is the types of the diagnostic modalities used. Echocardiography and multigated acquisition (MUGA) scan were the primary diagnostic tools used in clinical trials whereas their use was limited in the real-world lung cancer study that may cause inability to detect some forms of cardiotoxicities which can only be diagnosed with echocardiography or MUGA scan. As a result, even though the patient groups are from the same cancer diagnosis and treatments, the differences in monitoring strategy between clinical trials and the real-world lung cancer study may cause disparities in the detection of cardiotoxicity.

## Conclusion

Anti-cancer treatment-induced cardiotoxicity is a life-threatening adverse event. Phase 3 clinical trials play a role in understanding the efficacy of drugs in a selected group of patients. However, clinical trials do not appear to reflect the cardiovascular safety outcomes as a whole due to their strict eligibility criteria. Real-world studies have an ability to show the true incidence and types of cardiotoxicity more realistically than clinical trials as long as they have less strict inclusion and exclusion criteria.
